# Genetic Bases of Bicuspid Aortic Valve: The Contribution of Traditional and High-Throughput Sequencing Approaches on Research and Diagnosis

**DOI:** 10.3389/fphys.2017.00612

**Published:** 2017-08-24

**Authors:** Betti Giusti, Elena Sticchi, Rosina De Cario, Alberto Magi, Stefano Nistri, Guglielmina Pepe

**Affiliations:** ^1^Department of Experimental and Clinical Medicine, Section of Critical Medical Care and Medical Specialities, University of Florence Florence, Italy; ^2^Marfan Syndrome and Related Disorders Regional (Tuscany) Referral Center, Careggi Hospital Florence, Italy; ^3^Advanced Molecular Genetics Laboratory, Atherothrombotic Diseases Center, Careggi Hospital Florence, Italy; ^4^Center of Excellence for the Study at Molecular and Clinical Level of Chronic, Degenerative and Neoplastic Diseases to Develop Novel Therapies (DENOTHE), University of Florence Florence, Italy; ^5^Cardiology Service, Centro Medico Strumentale Riabilitativo (CMSR) Veneto Medica Altavilla Vicentina, Italy

**Keywords:** bicuspid aortic valve, genetics, high-throughput sequencing, next generation sequencing, gene, modifier gene, mendelian inheritance, multifactorial inheritance

## Abstract

Bicuspid aortic valve (BAV) is a common (0.5–2.0% of general population) congenital heart defect with increased prevalence of aortic dilatation and dissection. BAV has an autosomal dominant inheritance with reduced penetrance and variable expressivity. BAV has been described as an isolated trait or associated with syndromic conditions [e.g., Marfan Marfan syndrome or Loeys-Dietz syndrome (MFS, LDS)]. Identification of a syndromic condition in a BAV patient is clinically relevant to personalize aortic surgery indication. A 4-fold increase in BAV prevalence in a large cohort of unrelated MFS patients with respect to general population was reported, as well as in LDS patients (8-fold). It is also known that BAV is more frequent in patients with thoracic aortic aneurysm (TAA) related to mutations in *ACTA2, FBN1*, and *TGFBR2* genes. Moreover, in 8 patients with BAV and thoracic aortic dilation, not fulfilling the clinical criteria for MFS, *FBN1* mutations in 2/8 patients were identified suggesting that *FBN1* or other genes involved in syndromic conditions correlated to aortopathy could be involved in BAV. Beyond loci associated to syndromic disorders, studies in humans and animal models evidenced/suggested the role of further genes in non-syndromic BAV. The transcriptional regulator *NOTCH1* has been associated with the development and acceleration of calcium deposition. Genome wide marker-based linkage analysis demonstrated a linkage of BAV to loci on chromosomes 18, 5, and 13q. Recently, a role for *GATA4*/*5* in aortic valve morphogenesis and endocardial cell differentiation has been reported. BAV has also been associated with a reduced *UFD1L* gene expression or involvement of a locus containing *AXIN1*/*PDIA2*. Much remains to be understood about the genetics of BAV. In the last years, high-throughput sequencing technologies, allowing the analysis of large number of genes or entire exomes or genomes, progressively became available. The latter issue together with the development of “BigData” analysis methods improving their interpretation and integration with clinical data represents a promising opportunity to increase the disease knowledge and diagnosis in monogenic and multifactorial complex traits. This review summarized the main knowledge on the BAV genetic bases, the role of genetic diagnosis in BAV patient managements and the crucial challenges for the comprehension of genetics of BAV in research and diagnosis.

## Introduction

Bicuspid aortic valve (BAV) represents a common congenital heart defect (0.5–2.0% of the adult general population) (Prakash et al., 2014). Complications of BAV are: aortic regurgitation (13–30%), aortic stenosis (12–37%), infective endocarditis (2–5%), and dilatation of the thoracic ascending aorta (20–50%) (Cecconi et al., 2006; Della Corte et al., 2013; Masri et al., 2017); an increased risk of aortic dissection has been also documented in BAV population (De Cario et al., 2014; Verma and Siu, 2014; Masri et al., 2017). This is especially relevant as a large part of BAV patients encounter a valve damage requiring aortic surgery (the cumulative 25-years risk including 25% of aortic surgery and 53% of receiving valve replacement) and the high incidence of associated thoracic aortic aneurysm (TAA) formation (Michelena et al., 2011). Such BAV complications, if not early identified and appropriately managed, may indeed represent long-term health risks. The surveillance approach to the disease and the prophylactic surgical management of patients resulted in a survival rate similar to that of the comparable general population (Masri et al., 2017). A male preponderance (M:F = 3:1) has been noted among BAVs. The latter issue is of interest in relation also to the fact that BAV is frequent in the XO Turner syndrome where it may be the most common cardiac defect: >30% of patients with Turner syndrome had BAV (Miller et al., 1983; De Cario et al., 2014; Masri et al., 2017). Beyond the well-known hemodynamic bases of BAV, whereby an altered blood flow through the valve during its formation determines an abnormal cups formation, a role for genetics in contributing to the development of the disease has also been recognized (Longobardo et al., 2016). Although BAV usually represents an isolated feature, its association with other clinical manifestations suggestive of a syndromic disorder, has also been described (Prakash et al., 2014). Moreover, both mathematical algorithms and familial studies support the hypothesis that BAV could be heritable (Cripe et al., 2004; Loscalzo et al., 2007; Laforest and Nemer, 2012; Longobardo et al., 2016), even though the genetic bases of BAV largely remain to be elucidated. Actually, different molecular signaling pathways—involved in the formation of the outflow tract (OFT) and in the endocardial-mesenchymal transition (Laforest et al., 2011; Laforest and Nemer, 2012), migration of neural crest cells (Jain et al., 2011), or extracellular matrix (ECM) remodeling (Fedak et al., 2003)—have been demonstrated to be involved in aortic valve embryogenesis. Therefore, this issue supports the possible contribution of different loci in influencing the development of an abnormal valve formation and of other non-valvular complications associated with the disease. The present review will focus on up to date information concerning the genetic loci found to be associated with BAV (both syndromic and non-syndromic cases), and on the progressive advancements in massive parallel sequencing approaches able to generate a large volume of genetic data, thus representing a substantial challenge in contributing to faster elucidate the molecular bases of the disease, and to allow differential diagnosis for a better BAV patients management.

## Genetics of BAV

### What we know so far

The initial insight of a strong genetic component participating in the pathogenesis of this cardiac malformation has been provided by several studies revealing a high incidence of familial clustering. Early studies showed the aortic valve disease, probably resulting from BAV, to have a prevalence of 24% in families with more than one family member carrying the valve malformation (Glick and Roberts, 1994; Clementi et al., 1996). Shortly thereafter, a 9.1% prevalence of BAV was observed among 190 first-degree relatives in families screened by echocardiography (Huntington et al., 1997). More recent studies, that made use of a variance component methodology and a mathematical model, established the heritability of BAV up to 89% indicating the disease as almost entirely genetically determined (Cripe et al., 2004; Lewin et al., 2004; Freeze et al., 2016). Family data were consistent with an autosomal dominant pattern of inheritance with reduced penetrance and variable expressivity (Laforest and Nemer, 2012; De Cario et al., 2014).

Despite the well-established notion of a heritability associated with BAV, no single gene model exists that could explain the inheritance of this cardiac malformation. In fact, a role of many discrete genes with divergent inheritance pattern which might also act in combination as polygenic trait may be derived by the analysis of literature data (Laforest and Nemer, 2012; De Cario et al., 2014). Hence, during the last decades, a number of studies on animal models, together with various genetic and biochemical approaches, have been performed allowing the identification of a large number of genes suggested to be implied in BAV pathogenesis in its sporadic or syndromic presentation (Table 1). The products of these genes are represented by transcription factors, components of the ECM, and proteins involved in several signaling pathways regulating various cellular processes, such as proliferation or apoptosis, in cardiac tissues (Laforest and Nemer, 2012; De Cario et al., 2014; Freeze et al., 2016). Furthermore, over recent years, the wide heterogeneity in BAV has been suggested to be the result of a combination between genetic, functional and hemodynamic factors acting as modulators of the phenotype expression (Nistri et al., 2008, 2016; Conti et al., 2010; Della Corte et al., 2012; De Cario et al., 2014; Michelena et al., 2015; Fedak and Barker, 2016; Fedak et al., 2016; Longobardo et al., 2016; Masri et al., 2017). In most cases, BAV presents itself as an isolated trait but its association with other genetic syndromes, such as Andersen syndrome, Turner syndrome, William Beuren, Bosley-Salih-Alorainy, Athabascan Brainstem Dysgenesis syndromes is well established. In particular, it may occur as component of connective tissue disorders, such as familial TAA and dissection (FTAA/D), Marfan syndrome (MFS), Loeys-Dietz syndrome (LDS) and vascular Ehlers-Danlos syndrome (vEDS) (Duran et al., 1995). In fact, the identification of a syndromic condition in a BAV patient is relevant as it can affect the rate of progression to clinically evident disease and has led, for example, to the definition of distinct guidelines, with respect to aortic diameter thresholds, for elective surgery in MFS and BAV patients (Erbel et al., 2014; Nishimura et al., 2014). A recent study, carried out on a cohort of 257 MFS patients, allowed the unequivocal diagnosis of BAV in 12 patients (4.7%) revealing a prevalence of the cardiac valve malformation exceeding four times that observed in the general population screened by echocardiography (0.5%) (Basso et al., 2004; Nistri et al., 2005, 2012). In patients with LDS, BAV has been also noted as a clinical finding more prevalent (8%) than in the general population (Patel et al., 2017). These results suggest how aortic valve morphology in MFS patients should be better pursued during clinical examination. On the other hand, due to the wide range variability and severity of clinical manifestations in inherited connective tissue disorders patients often leading to a late definite diagnosis of syndromic phenotypes, such as MFS or LDS, the evaluation of the coexistence of a syndromic condition in BAV patients by an expert clinician/geneticist should be encouraged in order to better decide the follow-up and surgical timing. Genetic testing could therefore provide a valuable tool in order to detect not only genetic variants causative of BAV, furthering knowledge of the mechanisms underlying its pathogenesis, but also to predict and prevent the aforementioned BAV-associated complications and to identify at-risk asymptomatic family members.

**Table 1:** Genetic loci associated with BAV in humans and animal models.

**HUMANS**	
**NON-SYNDROMIC BAV**	**SYNDROMIC BAV**
*NOTCH1*	*FBN1* (Marfan syndrome)
*GATA5*	*TGFBR1/2* (Loeys-Dietz syndrome)
*GATA4* *ACTA2* Linkage loci on: Chr 15q Chr18q Chr 5q *UFD1L* *AXIN1/PDIA2* *ENG* *EGFR* *SMAD6*	*ACTA2* (Thoracic aortic aneurysm and dissection syndrome) *KCNJ2* (Andersen syndrome) 45 × 0 karyotype (Turner syndrome) Deletion of 1.5-1.8Mb region (7q11.3) including *CLIP2, ELN, GTF2I, GTF2IRD1*, and *LIMK1* (William Beuren syndrome) *HOXA1* (Bosley-Salih-Alorainy syndrome, Athabaskan brainstem dysgenesis syndrome) *COL3A1* (Vascular Ehlers Danlos syndrome)
**ANIMAL MODELS**	
*Notch1*	
*Gata5*	
*Nos3*	
*Nkx2.5*	

In 2012, in 2 out of 3 BAV/MFS patients undergoing mutation-screening analysis, genetic variants in *FBN1* gene were found (Nistri et al., 2012). Moreover, our group next identified for the first time pathogenetic *FBN1* gene (fibrillin 1, 15q21.1, OMIM^*^134797) mutations in patients with BAV and aortic dilation/aneurysm in whom MFS and other more severe type 1 fibrillinopathies were clinically excluded (Pepe et al., 2014). *FBN1* encodes a glycoprotein component of the ECM involved in the maintenance of elastic fibers and in the anchorage of epithelial cells to the interstitial matrix and a decreased *FBN1* mRNA or protein content has been demonstrated in a subgroup of BAV patients, suggesting this gene to be one of those possibly associated with BAV. In addition, it has been demonstrated that targeted deletion of *Fbn1* in mice recapitulates the vascular defects observed in MFS suggesting valve malformation to be the result of *FBN1* mutations (Pereira et al., 1997; Ng et al., 2004). An up-regulated transforming growth factor beta (TGF-ß) signaling was observed in these mice who showed mitral valve prolapse and died shortly after birth for aortic dissection as a consequence of aortic wall weakening (Ng et al., 2004). TGF-ß represents the key regulator of vascular matrix remodeling and vascular smooth muscle cells (VSMCs) activity, and a wide number of studies provided evidences of the association between the dysregulation of its signaling and aneurysm formation, including-BAV associated TAA (Kurtovic et al., 2011). Mutations in the TGF-β receptors, *TGFBR1* (transforming growth factor beta receptor, type 1, 9q22.33, OMIM^*^190181) and *TGFBR2* (transforming growth factor beta receptor, type 2, 3p24.1, OMIM^*^190182) have been described in MFS-like conditions and have been consistently associated with LDS (Loeys et al., 2005; Mátyás et al., 2006; Attias et al., 2009; Wei et al., 2016). A missense mutation in *TGFBR2* has been shown to segregate in a family with non-syndromic associated BAV and proximal aortic aneurysm which was identical to the one found in MFS patients who tested negative for mutation in *FBN1* (Girdauskas et al., 2011). More recent sequencing experiments on familial and isolated BAV cases failed to identify mutations in the two receptors suggesting their contribution to be probably very low in the overall BAV population (Arrington et al., 2008; Foffa et al., 2013; Bonachea et al., 2014b).

Mutations in *ACTA2* gene, encoding smooth muscle α-actin and known to be associated with familial TAA, have been also detected in patients with BAV (Jondeau and Boileau, 2012; Martín et al., 2017).

Moreover, further data from genetics studies (candidate gene as well as genome wide analyses) allowed to deepen the knowledge concerning the genetic bases of the disease, thus evidencing a role for other candidate loci not previously associated with syndromic BAV. *NOTCH1* (Notch, Drosophila, homolog of, 1, chr 9q34.3, OMIM^*^190198) mutations have been firstly associated with aortic valve abnormalities, such as aortic valve calcium deposition, suggesting their potential role in cardiac disease in humans. These studies suggested that mutations in this gene, identified in a small number of families, may represent the genetic basis for hypoplastic left heart syndrome in some patients (Garg et al., 2005). Further evidences of a *NOTCH1* haploinsufficiency as possible cause of aortic valve disease were provided by shortly subsequent studies in which targeted mutational analyses were performed, allowing the identification of not previously described missense mutations in patients with BAV and/or aortic aneurysms (Mohamed et al., 2006; McKellar et al., 2007; Foffa et al., 2013). These studies also unraveled the role of *NOTCH1* mutations in familial BAV as well as in approximately 4% of sporadic cases. Recently, genetic screening of 428 probands with left-sided congenital heart disease (LS-CHD) allowed the identification of 14 *NOTCH1* mutations (11 in familial and 3 in isolated cases), 10 out of 11 families and 1 out of 3 isolated cases showed BAV (Kerstjens-Frederikse et al., 2016). Interestingly, this datum suggests a higher prevalence of *NOTCH1* mutations among familial cases [11/148 (7%)] than among sporadic forms [3/280 (1%)] of LS-CHD. The Notch signaling pathway is highly conserved across species. *NOTCH1* encodes a large protein containing an extracellular domain with 36 tandem epidermal growth factor (EGF)-like repeats and three cysteine-rich Notch/LIN-12 repeats, an intracellular domain with six ankyrin repeats, and a transactivation domain (Artavanis-Tsakonas et al., 1999). Since the discovery of *NOTCH1* as a potential candidate gene underlying BAV formation, a number of studies carried out analysis of aortic valves in genetically engineered mice in an attempt to unravel the molecular mechanisms associated with valve development. Murine model studies indicate that targeted inactivation of Notch impairs endocardial epithelial-to mesenchyme transition *in vivo* and in explant assays. Notably, Notch1 null mice developed serious cardiac alterations which led them to an early death; one of these defects results in imperfect epithelial-to-mesenchymal transition (EMT). EMT, occurring in the developing cardiac valves, represents an important process determining the transition from primordial to mature valves (von Gise and Pu, 2012). In addition, mutations involving components of the Notch pathway affect the expression of some TGF-ß signaling members, indicating that Notch activity is needed for the normal functioning of several elements acting in these key intracellular processes (Timmerman et al., 2004). This is of particular importance since a large number of evidences support the key role of a dysregulated TGF-ß signaling in vascular matrix remodeling. BAV patients with dilated ascending aorta showed indeed a distinctive TGF-β pathway gene expression pattern with respect to dilated subjects with normal three leaflet valve, resulting, in turn, to modulate phenotypic heterogeneity of thoracic aneurysm in BAV. This is likely to represent one of the most frequent events implicated in aneurysm formation, which can occur in association with BAV in a more severe clinical phenotype. These findings provided initial insights on developmentally regulated EMT processes, including the occurrence of congenital cardiac valve abnormalities. Subsequently, Notch1 signaling has also been described as affecting molecular processes involved in aortic valve calcification. Engineered Notch1^+/−^ mice have in fact been shown to endure a >5-fold aortic valve calcification level with respect to their wild-type counterparts comparable for age and sex. These studies provided evidence of the repression mechanism, generally played by Notch1 in murine aortic valves *in vivo* and in aortic valve cells *in vitro*, on *BMP2* gene (bone morphogenic protein, 220p12.3, OMIM^*^112261), which partly stall the progression of aortic valve calcification (Nigam and Srivastava, 2009). In a more recent aortic valve calcification *in vitro* model, addition of Sox9 was found to prevent Notch signaling, implying Notch1 to act as a regulator of aortic valve calcification through a Sox-9-dependent pathway (Acharya et al., 2011). All together, these results strengthen the hypothesis according to which *NOTCH1* haploinsufficiency plays a fundamental part during the embryonic development of cardiac valves and also in maintaining their regular function in the mature heart. Its dysregulation may therefore predispose to BAV as well as other congenital cardiac malformations affecting both the left and right-sided cardiac OFTs in humans (Koenig et al., 2017).

Besides Notch, which appears to be critical for normal tricuspid formation, *Nos3* pathway has been implicated as a regulator of BAV formation in animal models. *Nos3* is expressed in endocardial cells of the heart and is shear-stress-dependent. *Nos3*^−/−^mice were reported to carry CHD in early studies (Lee et al., 2000). The relevance of *NOS3* (Nitric oxide synthase 3, 7q36.1, OMIM^*^163729) as possible regulator in BAV formation was also supported by other studies showing a significant reduction of its expression in patients with BAV (Aicher et al., 2007). *NOS3* activation is mediated by members of the GATA family of transcription factors (German et al., 2000), such as GATA5, encoded by *GATA5* gene (Gata-binding protein 5, 20q13.33, OMIM^*^611496).

Alongside *NOTCH1, GATA5* has been linked to BAV in humans as a number of studies reported several rare sequence variants of this gene in BAV and its associated aortopathy (Padang et al., 2012; Bonachea et al., 2014a; Shi et al., 2014), accounting for up to 4% of sporadic cases. *GATA5* has an essential role in cardiogenesis and aortic valve development as a mediator of cellular mechanisms participating in endocardial cell differentiation, some of these processes being regulated by *Bmp4, Tbx20* as well as *NOS3* and *NOTCH1* (Padang et al., 2012). A recent *Gata5* null mouse model showed partial penetrance of BAV with a prevalence of 26%; a decrease in *Nos3* expression was also observed in the endocardial cushions of the OFT together with a significant downregulation of Notch1 pathway (Laforest et al., 2011). In addition to that, a deleterious mutation in *NKX2.5* gene (Nk2 Homeobox 5; 5q35.1, OMIM^*^600584) that completely abolished its interaction with GATA5 was found to segregate with disease in a family with BAV and a small proportion (11%) of mice carrying cardiac homeobox *Nkx2*-*5* haploinsufficiency (Groenendijk et al., 2004) have been associated with a higher incidence of the disease. Homeobox protein Nkx-2.5 activity is critically required during cardiac morphogenesis and it's also involved in modulation of the ECM of the aorta due to its role as a regulator of the collagen type I availability (Ponticos et al., 2004). All together, these findings seem to support the notion of Notch1 and Nos3 pathways, mediated by GATA5, as possible relevant elements in BAV pathogenesis. Other genes encoding GATA family cardiac factors, such as *GATA4* (GATA-binding protein, 4; 8p23.1; OMIM^*^6005769) and *GATA6* (GATA-binding protein, 6; 18q11.2; OMIM^*^601656) were observed in human congenital heart defects (Garg et al., 2003; Lepore et al., 2006; Rajagopal et al., 2007; Hamanoue et al., 2009; Maitra et al., 2010). Recently, a low frequency noncoding variant 151 kb from *GATA4*, together with a missense mutation involving the same gene, showed an association with the BAV phenotype that reached genome wide significance. The case-control genome wide association study was carried out on 466 BAV patients and 4,660 controls, replicated in up to 1,326 cases and 8,103 controls. These identified variants are thought to affect cardiac valves development and increase the risk of cardiac malformations as they seem to disrupt some regulatory elements involved in *GATA4* expression during cardiac embryogenesis (Yang et al., 2017). Several other genes have been implicated in isolated BAV or in BAV-TAA (Laforest and Nemer, 2012; De Cario et al., 2014; Freeze et al., 2016). One of these is *UFD1L* (ubiquitin fusion degradation 1-like, 22q11.21, OMIM^*^601754), whose expression has been demonstrated to be down-regulated in BAV patients. Its product is a component of a multi-enzyme complex involved in the degradation of ubiquitin fusion proteins during embryogenesis, with an important role in the development of ectoderm-derived structures; it has been observed to be diminished in BAV patients (Mohamed et al., 2005).

Its important function in aortic leaflets formation indicates a possible role of *UFD1L* in BAV pathogenesis even if no causal relationship, but only an association, between mutations involving this gene and the cardiac malformation has been established so far. A missense mutation in the MH2 domain of the SMAD6 protein (p.Cys484Phe) in a man with BAV, aortic valve stenosis, and coarctation and calcification of the aorta was identified (Tan et al., 2012). Resequencing of the MH2 domain of SMAD6 gene (homolog of mothers against decapentaplegic, drosophila, 6 chr 15q22.31, OMIM ^*^602931) in a replication cohort consisting of 346 additional probands with a broad range of cardiovascular malformation phenotypes revealed another missense mutation (p.Pro415Leu) in an infant with BAV and moderate aortic stenosis. Gene network analysis identified haplotypes for *ENG* (endoglin, 9q34.11, OMIM^*^13195), a gene known to be important in heart valve formation, and for *AXIN1* (axis inhibitor 1; 16p13.3, OMIM^*^603816) and *PDIA2* (Protein Disulfide Isomerase Family A Member 2; 16p13.3, OMIM^*^608012) to be associated with BAV in a cohort of 68 probands (Wooten et al., 2010). AXIN1 is member of the Wnt pathway, which mediates TGF-β signaling and acts as a crucial regulator of both heart valve formation and cardiac neural crest development (Armstrong and Bischoff, 2004). The role of PDIA2 in heart valve formation is not yet unveiled.

**Figure 1:**
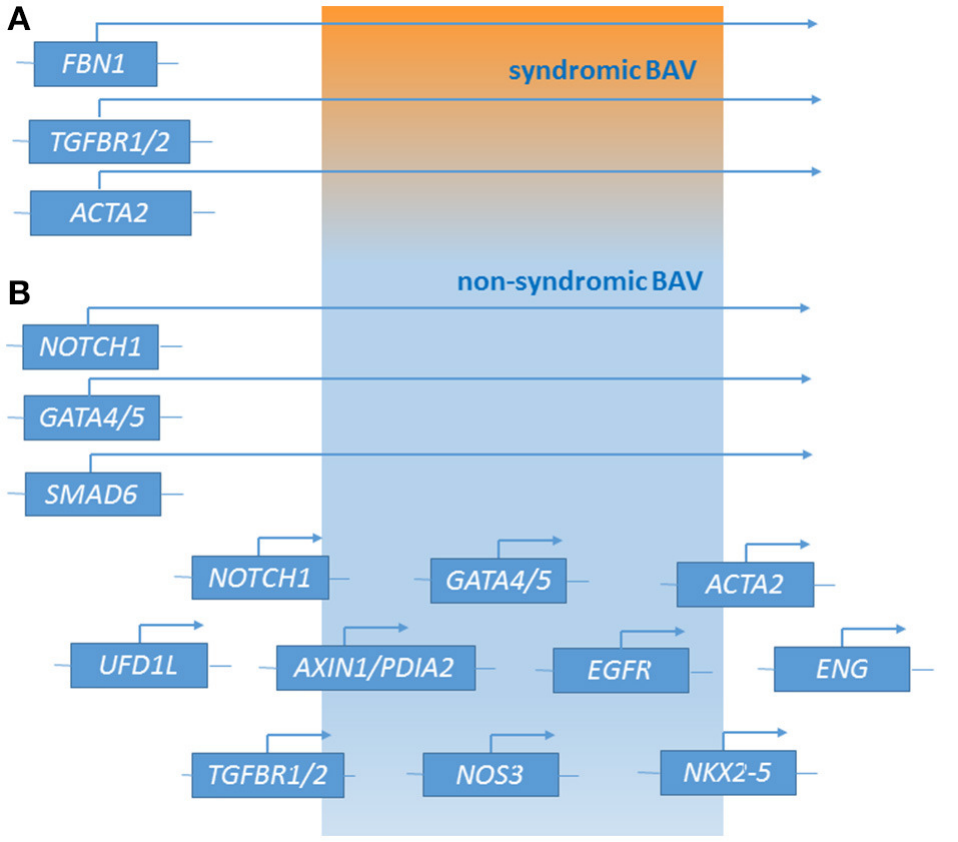
Genetic loci associated with syndromic BAV **(A)** and non-syndromic BAV **(B)** [effect of major genes or contribution of multiple minor loci (polygenic form of the disease)].

Recently, next generation sequencing (NGS) approaches on nine genes previously associated with BAV (*NOTCH1, AXIN1, EGFR, ENG, GATA5, NKX2-5, NOS3, PDIA2*, and *TGFBR2*) has been performed on 48 BAV patients (Dargis et al., 2016), allowing the identification of previously known potentially pathogenic variants in *AXIN1, EGFR, ENG, GATA5, NOTCH1*, and *PDIA2*. The most promising variants were subsequently evaluated in a case-control study showing men and women to carry some distinct genetic variants associated with BAV. These findings led to the hypothesis of the involvement of some gender-specific variants in BAV onset and advancement. Mutations in *ACTA2* (Actin, alpha 2, smooth muscle, aorta;10q23.3, OMIM^*^102620) were identified in 7 family members with aortic aneurysms and dissection, of whom 3 had BAVs, their aortic tissue displaying increased proteoglycans accumulation, fragmentation, loss of elastic fibers, and decreased numbers of smooth muscle cells, consistent with aortic wall degeneration. These findings could lead to the hypothesis of a unique pathogenetic basis for BAV-TAA patients (as BAV has been observed to occur more frequently in patients with TAA who have mutations in his gene) (Guo et al., 2007; Jondeau and Boileau, 2012) even if, at present, whether *ACTA2* mutations may cause BAV remains uncertain. Targeted NGS approach identified 31 rare non-synonymous, exonic variants classified as putative disease-causing changes by *in-silico* analysis in the 97 candidate genes. These study evidenced variants in 25 genes (*APC, AXIN2, FLT1, GATA4, GLI1, JAG1, MCTP2, MSX1, NFATC1, NOS1, NOTCH2, NOTCH3, PAX6, PIGF, PPP3CA, PTCH1, PTCH2, SLC35B2, SNAI3, SOX9, TBX5, VEGFB, VEGFC, WNT4, and ZNF236*) not previously associated with human BAV (Bonachea et al., 2014b). Finding these genetic variants in index cases did not imply a definitive association of these genes with the BAV phenotype, thus requiring further functional analyses and segregation data in families. Ultimately, BAV seems to display a substantial genetic heterogeneity, suggesting the role of many discrete genes in its pathogenesis, which is challenging for researchers whose aim is to discover genetic variants causative of BAV as well as patients at risk to develop the most feared BAV-associated complications (Figure 1). The aforementioned animal studies provided evidences of a combination between multiple genetic variants acting as a burden and epigenetic plus environmental factors. This complex array of genetic and non-genetic factors may be responsible for the extremely variable phenotypic expression of BAV. This is especially relevant considering those conditions that often accompany BAV as TAA/D.

Due to the evidence that BAV and TAAD frequently occur together, we might hypothesize that BAV genetic profile may be determined by an additive contribution of many different genetic variants increasing both the risk for BAV occurrence and for its complications (Prakash et al., 2014). Therefore, successful gene discovery may help in situations in which the identification of the leading factor, hemodynamic or genetic, playing a relevant role in the disease development, determines different surgical decisions (Padang et al., 2012). In fact, wherever a genetic alteration is identified as the dominant factor, this may lead to a more aggressive disease phenotype and earlier aortic intervention (Erbel et al., 2014; Nishimura et al., 2014). To that purpose, large families with inherited predisposition to BAV currently represent the most promising opportunities for the consolidation of suspected and novel discovery of gene association even considering (1) the known difficulties in recruiting well clinically evaluated relatives, together with (2) the reduced penetrance and variable expressivity associated with the BAV phenotype. Even and above all, in the era of high-throughput technologies, segregation analyses represent the gold standard approach to identify causative mutations in genes of distantly related subjects, also providing and optimizing the statistical power in genotype-phenotype correlations (Michelena et al., 2014). As thousands of cases are needed to identify substantial genetic contributors, constant advances in NGS technologies provide an unprecedented opportunity to unravel the genetic complexity of BAV and its associated aortopathies (Andreassi and Della Corte, 2016). Targeted NGS of a carefully selected part of the genome (a specific set of genes relevant to a disease phenotype) produces a more manageable data set compared with broader approaches, making analysis easier and faster (Wooderchak-Donahue et al., 2015). This kind of approach may lead to the identification of novel genetic variants whose biological role in BAV is yet to be determined; these variants could be tested for a possible association with the disease also when assessed together in intracellular signaling pathways (Michelena et al., 2014). Nevertheless, it should be considered that the comprehension of the pathogenesis of BAV could be further supported by different mechanisms not addressed in this review. In fact, beyond the presence of mutations in exons or splice site consensus regions of protein-coding genes, mutations in introns and regulatory 5′ promoter and UTR regions, intergenic variants, as well as epigenetic mechanisms involving miRNAs expression profiles alterations or DNA methylation and histone modifications may also have a role in BAV development.

### High-throughput sequencing (HTS) technologies:

The achievement of information on the genetic bases of BAV has been allowed by the progressive acquisition of novel technologies able to produce a large volume of data. Actually, first studies were based on the evaluation of the heritability of BAV, thus providing information on the contribution of genetics to this phenotypic trait. To that end, family-based approach has revealed useful in identifying loci segregating with the disease. Afterwards, genome wide association studies (GWAS) investigating a wide number of genetic variants in genes supposed to be associated with the disease added a further support in identifying novel candidate loci (Wang et al., 2009; De Cario et al., 2014; Freeze et al., 2016). Although a number of information on genetic contribution to BAV disease have been obtained through these approaches, most remains to be discovered and understood concerning genetics of BAV. At this purpose a relevant contribution in deepening the molecular bases of BAV may derive from high-throughput sequencing (HTS) technologies also identified as next generation sequencing (NGS), allowing the parallel analysis of large number of genes or entire exomes or genomes. Advancements in DNA sequencing technologies led to the progressive availability of several platforms, each one exhibiting differences in detection methods and throughput, in order to cover needs ranging from target genes panel sequencing approach to exomes/genomes analyses. The resulting availability of large volume of data determined also the development of novel “BigData” analysis methods, able to integrate genetic and clinical data, thus representing a promising opportunity, at present and in the next few years, to significantly increase the knowledge on the genetic bases of BAV. Actually, since 2008, the diffusion of HTS allowed reduction in costs per run and time of analysis, thus raising interest in the use of HTS approach both as a research and clinical tool (https://www.genome.gov/27541954/dna-sequencing-costs-data/; Goodwin et al., 2016; Magi et al., 2017).

Each platform exhibits specific chemistry and detection methods, thus differently contributing to the overall performance of the sequencing approach and then differently adapting to the needs and peculiarities of research and diagnostics.

The first HTS technology to be widespread is represented by the Roche 454 (GS FLX) platform (Roche Diagnostics; Table 2), released in 2005 (Margulies et al., 2005) and just discontinued (Levy and Myers, 2016), a sequencing-by-synthesis (SBS) approach based on *single nucleotide addition* (SNA) sequencing principle. In this approach clonal template populations are generated after sample DNA fragmentation followed by ligation with an oligonucleotide adaptor, complementary to an oligonucleotide fragment immobilized on the surface of a capture bead, and emulsion PCR (emPCR), carried out in aqueous droplets (Shendure et al., 2005). Consequently, multiple copies of the same DNA sequence will cover each capture bead. The beads are next arrayed in the wells of a fiber optic slide (PicoTiterPlate) for the pyrosequencing reaction (Leamon et al., 2004).

**Table 2:** High-Throughput sequencing (HTS) platforms characteristics.

**Platform**	**Chemistry**	**Read length (bp)**	**Throughput**	**Error rate (%)**	**Primary error type**
ROCHE 454^*^					
GS FLX Titanium XLR70	*Sequencing-by-synthesis*	Up to 600	450 Mb	1	indel
GS FLX Titanium XL+	*Sequencing-by-synthesis*	Up to 1000	700 Mb	1	indel
**ION TORRENT^∧^**					
Ion PGM 314	*Sequencing-by-synthesis*	200	30–50 Mb	1	indel
		400	60–100 Mb		
Ion PGM 316	*Sequencing-by-synthesis*	200	300–500 Mb	1	indel
		400	600 Mb-1 Gb		
Ion PGM 318	*Sequencing-by-synthesis*	200	600 Mb–1 Gb	1	indel
		400	1–2 Gb		
Ion Proton	*Sequencing-by-synthesis*	Up to 200	Up to 10 Gb	1	indel
Ion S5 520	*Sequencing-by-synthesis*	200	600 Mb–1 Gb	1	indel
		400	1.2–2 Gb		
Ion S5 530	*Sequencing-by-synthesis*	200	3–4 Gb	1	indel
		400	6–8 Gb		
Ion S5 540	*sequencing-by-synthesis*	200	10–15 Gb	1	indel
**ILLUMINA^∧^**					
MiniSeq	*Sequencing-by-synthesis*	Up to 150	1.65–7.5 Gb	<1	substitutions
MiSeq	*sequencing-by-synthesis*	Up to 300	540 Mb–15 Gb	0.1	substitution
NextSeq 500/550	*Sequencing-by-synthesis*	Up to 150	16.25–120 Gb	<1	substitution
HiSeq 2500	*Sequencing-by-synthesis*	Up to 250	9–1000 Gb	0.1	substitution
HiSeq 3000/4000	*Sequencing-by-synthesis*	Up to 150	105–1500 Gb	0.1	substitution
HiSeqX	*Sequencing-by-synthesis*	Up to 150	1600–1800 Gb	0.1	substitution
PACIFIC BIOSCIENCES^*^					
RSII	*Single-molecule real-time long-reads*	~20,000	500 Mb–1 Gb	13 (single-pass); ≤1% circular consensus read^*^	indel
Sequel	*Single-molecule real-time long-reads*	8,000–12,000	3.5–7 Gb	–	–
**OXFORD NANOPORE^‡^**					
Mk1 MinION	*Single-molecule real-time long-reads*	Up to 1 Mb^*^	Up to 20 Gb^∧^ (1-D yield)	–	–

* Goodwin et al., 2016;

∧ Goodwin et al., 2016 and manifacturers's data (http://www.thermosisher.com/it/en/Home/brands/ion-torrent.html);

^†^Goodwin et al. (2016) and manifacturers's data manifacturers's data (http://www.illumina.com);

‡ *Manifacturers's data (http://www.nanoporetech.com)*.

Among SBS approaches, Ion Torrent and Illumina platforms are currently used (Table 2). Ion torrent technology also shares with the Roche 454 system the SNA principle and the emulsion PCR step to achieve the clonal amplification of the DNA template, whereas Illumina technology foresees a solid-phase bridge amplification and a *cyclic reversible termination* (CRT) approach (Goodwin et al., 2016).

Ion Torrent platform, although representing a very similar technology with respect to Roche 454, it does not rely on the optical detection of incorporating nucleotide through imaging technology, but it is based on a semiconductor sequencing approach, as proton release detection during nucleotide incorporation may be permitted by the use of ion sensors. Therefore, this approach requires smaller instrument size and may result in higher speed of sequencing analysis and lower costs with respect to Roche 454 (Metzker, 2010; Liu et al., 2012; van Dijk et al., 2014). Ion Torrent platform appears particularly useful for targeted sequencing approached in which a panel of specific genes is analyzed for diagnosis and research purpose.

Differently from previously described sequencing approaches, Illumina technology is based on the “bridge amplification” principle, in which DNA fragments, ligated to oligonucleotide adapters complementary to Illumina flow-cell anchors, are amplified *in-situ* on the flow-cell surface. This amplification step relies on arching of the captured DNA strand and its subsequent hybridization to an adjacent anchor oligonucleotide, thus contributing to generate clonally amplified clusters, each one including thousands of clonal molecules (Voelkerding et al., 2009). Afterwards, sequencing is performed through CRT approach, based on the use of fluorescent reversible dye terminators, in which the presence of a chemical modification of the ribose at the 3′-hydroxyl position of the nucleotide, allows a single-base extension during each sequencing cycle. After image acquisition, the reversible dye terminators are unblocked and the next cycle may be performed (Voelkerding et al., 2009). At present, Illumina platform, with its wide range of instruments with different through-put and lower costs, is the widespread HTS technology in research as well as diagnosis laboratories.

Although advancements in generating longer reads are currently being developed, the abovementioned platforms achieved significant results in providing sequencing data, with different strengths and weaknesses depending of each platform characteristics.

The Illumina technology (read length 35–300 bp) is shared by a large number of instruments, ranging from lower throughput benchtop units, such as MiniSeq (<7.5 Gb) or MiSeq v3 (<15 Gb), to ultra-high-throughput instruments, such as HiSeq × (800–900 Gb per flow cell) (Goodwin et al., 2016), useful for providing whole genome sequencing data at a population level. The different chemistry (CRT approach) of Illumina technology contributes to make it less susceptible to homopolymers errors, as observed in other platforms (Roche 454 and Ion Torrent). Nevertheless, although an overall accuracy of >99.5% could be recognized for this approach, under-representation in both AT- and GC-rich regions and substitution error have been observed (Bentley et al., 2008; Dohm et al., 2008; Harismendy et al., 2009; Minoche et al., 2011; Nakamura et al., 2011). The availability of different Illumina platforms contributes to render this technology suitable for a large number of applications, ranging from target gene panel/whole exome/whole genome sequencing to epigenomics (Park, 2009) as well as transcriptomics (Wang et al., 2009) applications.

If compared with Illumina technology, both Roche 454 and Ion Torrent HTS sequencing platforms generate higher length reads (up to 1,000 and 400 bp, respectively), thus offering a better efficiency in providing information concerning complex /repetitive DNA regions. Nevertheless, although their overall error higher rate in non-homopolymeric DNA regions could be considered comparable with that of other platforms, a higher prevalence of false positives in insertion/deletion (indel) variants detections is more commonly observed (Loman et al., 2012; Forgetta et al., 2013).

Over the last years, further advancements in HTS technologies have allowed to improve read length, thus possibly overcoming limitations of short read sequencing, such as *de-novo sequencing* and detection of structural features of the genome, by spanning these regions with a single continuous read. Actually, a wider resolution of genomic variations might be achieved in the presence of a complete, reference-free, genome assembly. The generation of longer reads could also be useful in the transcriptomics field, in order to correctly discern gene isoforms by spanning an entire mRNA transcript (Goodwin et al., 2016). Moreover, the short reads (100–50 bp) generated by previously mentioned platforms are not enough adequate to resolve complex genomic structures, such as long repetitive elements, copy number alterations and structural variations that could play a crucial role in the pathogenesis of BAV as well as other diseases in all the fields of medicine (Magi et al., 2017).

To date, long-read sequencing is primarily based on a *single-molecule real-time* (SMRT) sequencing approach, in which the generation of a clonally amplified DNA fragments population is no longer required in the sequencing protocol. The most widely adopted SMRT approach is that developed by Pacific Biosciences (Table 2) (read length average >14 kb) (Eid et al., 2009; Reuter et al., 2015; Levy and Myers, 2016), which foresees the use of specialized flow cells including thousands of picolitre wells (*zero-mode waveguide*, ZMW), at whose transparent bottom a polymerase is fixed, thus allowing labeled dNTP incorporation on each single molecule template. After the fluorescence signal is recorded by the instrument imaging system, the enzyme removes the fluorophore from the nucleotide and permits the next labeled dNTP to be added. Of interest, the present approach uses a unique circular DNA template, thus allowing to consecutively sequence the same DNA molecule several times (Eid et al., 2009; Loomis et al., 2013).

The use of nanopores in sequencing technologies was widely discussed from 1996 (Kasianowicz et al., 1996; Branton et al., 2008), thus providing new challenges in the sequencing technology field.

In 2014 the Oxford Nanopore Technology (ONT) developed a handheld sequencer based on nanopore sequencing technology, the MinION system (Table 2), able to directly detect the DNA base composition of a native single strand DNA molecule. It is a disposable device containing a sensor chip, application specific integrated circuits (ASIC) and nanopores that are needed to perform single molecule DNA sequencing experiments. The DNA sequencing with Nanopore instrument relies on the conversion of electrical signal of nucleotides passing through a nanopore in a membrane between two electrolytes. The experimental protocol foresees the use of a leader-hairpin library structure, with the reverse strand linked by a hairpin adaptor to the forward strand, thus allowing, when DNA passes throughout the pore, the consecutive sequencing of both strands (2D-reads) (Goodwin et al., 2016). Data from literature showed that the current MinION platform is able to generate approximately 100 Mb of data per 16-h run, with an average read length of about 6 kb (Ashton et al., 2015). Moreover, the ONT PromethION platform, more recently released (Levy and Myers, 2016; Gigante, 2017), represent an ultra-high-throughput platform including 48 individual flow cells, each one including 3000 nanopores, thus providing a large volume of data (about 2–4 Tb) in a 2-day run.

A recent study showed a significant improvement in *de novo* genomes' assembly and in exploration of structural variants by nanopore technology application (Magi et al., 2017). This approach might represent a further challenge to improve the comprehension of genetics of BAV.

## Conclusions and future perspectives

During the last decades, the hypothesis of an underlying genetic contribution in the pathogenesis of BAV has been supported by a growing number of evidences resulting from both human and animal models, even though no precise association between specific genes and the disease has been established in the majority of cases.

As other vascular and cardiovascular diseases, beside the mendelian inheritance observed in some families due to mutations in some genes (e.g., *NOTCH1*) with a strong pathogenetic effect, in the large part of BAV patients the disease is likely to have a multifactorial nature. The latter condition is the result of complex interactions among genetic alterations (from common to rare genetic variants and chromosomal abnormalities), hemodynamic shear stress (produced by the abnormal leaflets), and other environmental and stochastic factors. To further complicate the pathogenic complexity, BAV often accompanies syndromic disorders, such as MFS or LDS thus being difficult to distinguish whether the pathogenetic variants determining the syndromic picture are also responsible for BAV or they represent two different pathogenetic conditions due to genetic variants in different genes (Figure 1). The availability of high-throughput sequencing (HTS) technologies, enabling rapid and relatively cheap analyses of panel of genes or whole exome/genome, plays a fundamental role in achieving a better comprehension of the genetic bases of isolated and syndromic BAV. In this context, alongside the study of large cohorts of probands, the definite contribution deriving from functional analyses and segregation data in families should be encouraged.

At present, from a diagnostic point of view, performing differential genetic diagnosis through HTS techniques in order to exclude syndromic traits in BAV patients with suggestive manifestations should be considered.

Indeed, the greatest interest in obtaining more insights on the genetic contributors of aortic valve malformations lies in the opportunity to translate this knowledge in the clinical practice, aimed to predict BAV most feared complications (mainly aortic aneurysm and dissection), and make decisions on the best options and timing for aortic surgery (based on individualized genetic risk profiles) as well as on global patients management.

## Author contributions

All the Authors substantially contributed to (1) the conception or design of the work, acquisition a revision of literature data; (2) drafting the work or revising it critically for important intellectual content; (3) final approval of the version to be published; AND All Authors agree to be accountable for all aspects of the work in ensuring that questions related to the accuracy or integrity of any part of the work are appropriately investigated and resolved.

### Conflict of interest statement

The authors declare that the research was conducted in the absence of any commercial or financial relationships that could be construed as a potential conflict of interest.
